# Risk factors for *Pneumocystis* pneumonia with acute respiratory failure among kidney transplant recipients

**DOI:** 10.1186/s12882-023-03071-y

**Published:** 2023-02-09

**Authors:** Hak-Jae Lee, Hyun-Wook Kwon, Jong-Kwan Baek, Chan-Hee Park, Hye-Kyung Seo, Suk-Kyung Hong

**Affiliations:** 1 Division of Acute Care Surgery, Department of Surgery, University of Ulsan College of Medicine, Asan Medical Center, 88 Olympic-ro, 43-gil, Songpa-gu, 05505 Seoul, Korea; 2 Division of Kidney and Pancreas Transplantation, Department of Surgery, College of Medicine, University of Ulsan, Asan Medical Center, Seoul, Korea

**Keywords:** Kidney transplantation, Respiratory insufficiency, Pneumonia, Pneumocystis, Graft rejection

## Abstract

**Purpose:**

One of the rare life-threatening fungal infections is pneumocystis pneumonia (PCP). Immunocompromised patients are the main vulnerable population. We investigate the risk factors associated with the development of severe PCP infection with acute respiratory failure after kidney transplantation.

**Materials and methods:**

This is a retrospective, single-center, case-control study. PCP patients who are kidney transplant recipients and required high-flow oxygen support or mechanical ventilation between March 2009 and February 2017 were included in the study. The comparison was conducted between the non-severe and severe PCP groups. To identify associated risk factors, we performed univariate and multivariate logistic regression.

**Results:**

Among the total 2,330 kidney transplant recipients, 50 patients (2.1%) were diagnosed with PCP. Of these, 27 patients (54.0%) had severe PCP and 7 patients (14.0%) died, all of them were severe PCP patients. In the severe PCP group, the time from transplantation to PCP diagnosis (23.4 ± 24.9 months vs. 13.7 ± 9.9 months, p = 0.090) was insignificantly faster than in the non-severe PCP group. According to multiple logistic regression analysis, the significant risk factors associated with severe PCP were as follows, age (odds ratios (OR) 1.07; 95% confidence intervals (CI): 1.01–1.13; p = 0.027), time from transplantation to PCP diagnosis (odds ratios (OR) 0.92; 95% confidence intervals (CI): 0.86–0.99; p = 0.024), lymphopenia (OR 6.48; 95% CI: 1.05–40.09; p = 0.044), and history of acute rejection within 1 year (OR 8.28; 95% CI: 1.29–53.20; p = 0.026).

**Conclusion:**

Patients who have lymphopenia at the time of hospital admission or have been recently treated with acute rejection are more likely to progress to severe PCP, requiring intensive monitoring and aggressive treatment.

## Introduction

*Pneumocystis jiroveci* is the responsible organism for Pneumocystis pneumonia (PCP), which mainly infects immunocompromised patients. In the past, the main vulnerable group was patients with human immunodeficiency virus (HIV). However, the increased use of chemotherapy or immunosuppressants in solid organ transplant recipients leads to an increase in PCP incidence in HIV-negative patients [1–3].

With recent development in immunosuppressants, solid organ transplantation has increased. However, PCP occurred most commonly in kidney transplant recipients among solid organ transplants [4, 5]. Many investigations addressed the clinical outcomes of PCP patients with previous kidney transplantation and the associated risk factors. Old age, cytomegalovirus (CMV) co-infection, type of transplantation, allograft rejection, lymphopenia, and type of immunosuppressant were reported as risk factors for PCP [6–10].

Trimethoprim-sulfamethoxazole (TMP-SMX), as a prophylaxis treatment, had significantly decreased the incidence of PCP after kidney transplantation, but mortality rates are still high [5, 9, 11], and PCP incidence ranged from 3.6% to 7% [12–14].

The main cause of death among patients with PCP was pneumonia aggravation. Despite the development of intensive care treatments such as extracorporeal membrane oxygenation (ECMO), mortality rates in the presence of respiratory failure have been reported to be as high as 30–50% [15, 16].

The main feature of HIV-negative patients infected with PCP is acute onset hypoxic respiratory failure [17, 18]. Patients exhibiting mild symptoms often require a shorter treatment duration and make a quick recovery on antibiotics. Although, PCP patients with acute respiratory failure usually have a poor prognosis [4, 19]; the mortality rates are high, and the incidence of patients requiring mechanical ventilation ranges between 40 and 50% [1, 3, 15, 16, 19, 20]. Therefore, these patients require aggressive treatment. Few studies have assessed the risk factors of patients with PCP with acute respiratory failure requiring mechanical ventilation. We investigate risk factors associated with the development of severe PCP infection with acute respiratory failure after kidney transplantation.

## Materials and methods

### Patients and data Collection

**Fig. 1:**
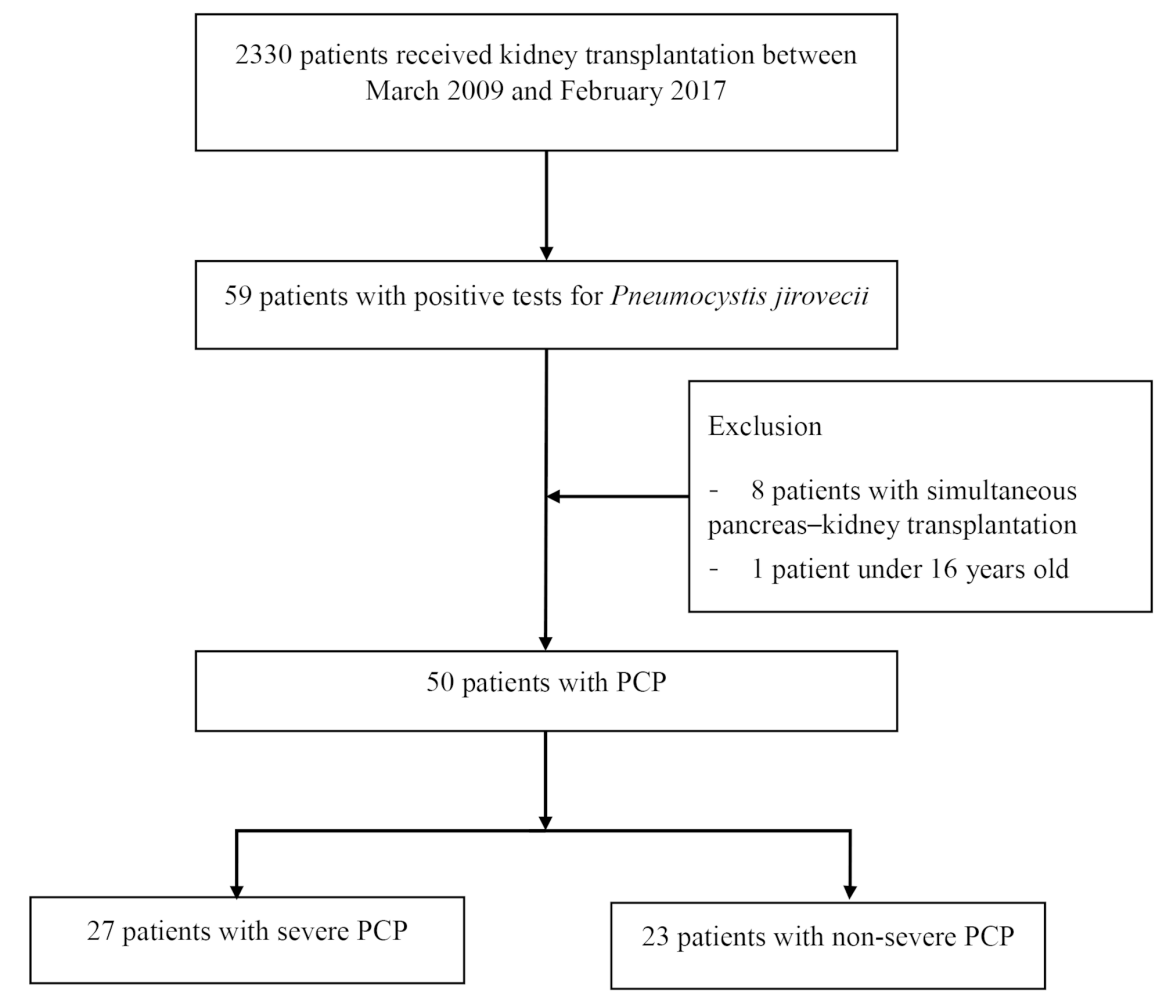
Patients enrolled in this study

This retrospective study included 2,330 patients who received kidney transplantation at Asan Medical Center between March 2009 and February 2017. Of these, 59 patients were diagnosed with PCP. Nine patients were excluded; eight underwent simultaneous pancreas-kidney transplantation, and one was under the age of 16. As a result, 50 patients were included for analysis (Fig. 1). Later, we retrospectively reviewed medical records and radiographs of the patients. Collected patient information included demographics, PCP prophylaxis, kidney donation type, operation type (living vs. deceased), acute rejection, and time from symptom onset to hospital admission. Clinical data such as laboratory results were also collected, including white blood cell count and total lymphocyte count (TLC), and the presence of combined infection, such as BK virus and CMV. The institutional review board of Asan Medical Center approved the conduction of this study (no.2020 − 0737).

### Definitions

Patients with clinically suspicious symptoms or radiologic finding such as diffuse ground-glass opacity received confirmation of PCP diagnosis with polymerase chain reaction (PCR) using bronchoalveolar lavage (BAL) fluid samples. Severe PCP was defined as patients requiring mechanical ventilation or high-flow oxygen support with a PaO2/FiO2 ratio of 200 or less at the time of hospital admission.

All patients were administered TMP-SMX (80–400 mg) daily for 6 months post kidney transplantation according to PCP prophylaxis protocol. TMP-SMX prophylaxis failure was defined as cases in which medication was not administered due to adverse drug reactions or decreased compliance ≤ 6 months after transplantation. In case of suspected acute rejection, a kidney biopsy was conducted. PCP prophylaxis was administered up to 6 months after rejection, so we defined acute rejection as any case with a history of treatment for acute rejection within one year before the PCP diagnosis. CMV combined infection was defined as patients receiving anti-viral treatment due to CMV viremia or CMV infection of other organs, such as gastritis. TLC was measured at the time of PCP diagnosis, and < 1,000 mm^3^ was defined as lymphopenia.

In patients diagnosed with PCP, TMP-SMX was used as the first-line antibiotic. TMP was administered in 3 or 4 divided doses of 15–20 mg/kg/day. As side effects of TMP-SMX, severe hyperkalemia, progression of acute kidney injury, and gastrointestinal intolerance such as nausea and vomiting occurred and the change to second-line antibiotics was defined as an adverse reaction. Treatment failure was defined as a case of changing to second-line antibiotics due to worsening of PCP despite the use of first-line antibiotics (TMP-SMX). As second-line antibiotics, 600 mg of IV (intravenous) clindamycin was given every six hours and oral primaquine 30 mg was administered once a day. In the case of severe PCP, adjunctive corticosteroid was given for therapeutic purposes. IV methylprednisolone (MPD) 30 mg was administered twice a day for 5 days, then 30 mg once a day for 5 days, and then IV MPD 15 mg was given once a day for 11 days.

Rituximab, which is a monoclonal antibody against CD20, is used in some KT recipients for pre-transplant desensitization or acute rejection treatment after transplant. Some investigations demonstrated an increased risk of PCP infection after rituximab administration. In this research, the use of rituximab was also included as a predictor of risk factors for respiratory failure. Additionally, rituximab was used in some patients (7 patients) for the treatment of acute rejection. And lymphocytes decreased in some patients, but all patients recovered to the normal range. Patients included in this study were patients who recently developed lymphocyte depletion during PCP infection.

### Statistical analysis

Student’s *t*-test and the Mann–Whitney U test were employed to examine the difference between continuous variables. Kruskal–Wallis test was used to compare various groups. Univariate and multivariate logistic backward regression analyses were conducted to predict the risk factors of respiratory failure in PCP patients after kidney transplantation based on clinical data, using odds ratios (ORs) and 95% CIs. P-values < 0.05 were considered statistically significant. R software was used for carrying out all statistical tests (version 4.0.3; R Foundation for Statistical Computing, Vienna, Austria; http://www.R-project.org).

## Results

Among the 2,330 kidney transplant recipients, 50 patients (2.1%) were treated with PCP. The mean age of the patients was 47.22 ± 14.35 years. Living donor kidney transplantation accounted for 40 (80.0%) total cases. 29 patients (58.0%) were infected with PCP within 1 year after transplantation. 19 patients (38.0%) were treated for acute rejection within 1 year of PCP diagnosis (Table 1).

**Table 1:** Demographic and clinical characteristics of patients with PCP according to respiratory failure

		Total (n = 50)	Non-severe PCP (n = 23)	Severe PCP (n = 27)	P-value
**Age (years)**		47.22 ± 14.35	41.4 ± 14.7	52.2 ± 12.3	0.007*
**Male (n, %)**		28 (56.0%)	13 (56.5%)	15 (55.6%)	1.000
**BMI**		21.81 ± 3.33	20.4 ± 2.8	23.0 ± 3.3	0.005*
**Underlying disease** Diabetes mellitus Hypertension Tuberculosis Hepatitis B virus Hepatitis C virus		10 (20.0%) 44 (88.0%) 3 (6.0%) 4 (8.0%) 1 (2.0%)	5 (21.7%) 21 (91.3%) 1 (4.3%) 2 (8.7%) 0	5 (18.5%) 23 (85.2%) 2 (7.4%) 2 (7.4%) 1 (3.7%)	1.000
**Causes of transplantation** Hypertension Diabetes mellitus Glomerulonephritis Polycystic kidney disease Unknown		6 (12.0%) 11 (22.0%) 13 (26.0%) 4 (8.0%) 16 (32.0%)	3 (13.0%) 3 (13.0%) 8 (34.8%) 0 9 (39.1%)	3 (11.1%) 8 (29.6%) 5 (18.5%) 4 (14.8%) 7 (25.9%)	0.139
**Type of kidney donation** Living related donor Living unrelated donor Deceased donor		25 (50.0%) 15 (30.0%) 10 (20.0%)	14 (60.9%) 6 (26.1%) 3 (13.0%)	11 (40.7%) 9 (33.3%) 7 (25.9%)	0.324
**Type of immunosuppressant** Tacrolimus Cyclosporine Mycophenolate mofetil Sirolimus		35 (70.0%) 15 (30.0%) 41 (82.0%) 2 (4.0%)	16 (69.6%) 7 (30.4%) 18 (78.3%) 2 (8.7%)	19 (70.4%) 8 (29.6%) 23 (85.2%) 0	1.000
**Co-infection** Cytomegalovirus BK virus		18 (36.0%) 10 (20.0%)	7 (30.4%) 4 (17.4%)	11 (40.7%) 6 (22.2%)	0.645 0.943
**ABO incompatible**		13 (26.0%)	6 (26.1%)	7 (25.9%)	1.000
**Re-transplantation**		3 (6.0%)	0	3 (11.1%)	0.293
**Incidence of TMP/SMX** **Prophylaxis failure**		12 (24.0%)	4 (17.4%)	8 (29.6%)	0.498
**Time from onset of symptom to hospital admission** (days)		7.3 ± 6.0	9.1 ± 7.6	5.8 ± 3.8	0.063
**Time from transplantation to PCP** (months)		18.1 ± 18.8	23.4 ± 24.9	13.7 ± 9.9	0.090
**Time from discontinuation of TMP/SMX to PCP** (months)		8.9 ± 14.0	12.8 ± 19.9	5.6 ± 2.7	0.101
**HLA-A, B, DR mismatch**		2.9 ± 1.5	2.3 ± 1.1	3.4 ± 1.7	0.011*
**Total lymphocyte count** (mm^3^) (normal range: 800-4,000 /mL)		755.0 ± 421.5	970.7 ± 408.3	571.2 ± 342.7	0.001*
**History of acute rejection treatment within 1 year**		19 (38.0%)	6 (26.1%)	13 (48.1%)	0.190
**Use of rituximab**		19 (38.0%)	6 (26.1%)	13 (48.1%)	0.190
**Change of second-line** **antibiotics**		25 (50.0%)	10 (43.5%)	15 (55.6%)	0.570
**Use of adjunctive** **corticosteroid**		34 (68.0%)	10 (43.5%)	24 (88.9%)	0.002*
**Length of hospital stay** (days)		39.94 ± 68.38	17.1 ± 9.9	59.4 ± 88.8	0.001*
**Mortality**		7 (14.0%)	0	7 (25.9%)	0.026*

* p-values of < 0.05 were considered statistically significant

BKV, BK virus; BMI, body mass index; CMV, cytomegalovirus; HLA, human leukocyte antigen; PCP, pneumocystis pneumonia; TMP/SMX, trimethoprim/sulfamethoxazole

A total of 27 patients (54.0%) had an acute respiratory failure that required high-flow oxygen support because the PaO2:FiO2 ratio was less than 200. Of these, 18 patients (36.0%) were treated with mechanical ventilation. Five patients (10.0%) received extracorporeal membrane oxygenation (ECMO). And seven patients (14.0%) died. All patients were initially treated with TMP-SMX. Of these, 25 patients (50.0%) were transferred to secondary antibiotics (clindamycin and primaquine). 13 patients (26.0%) changed antibiotics due to primary treatment failure, and 12 patients (24.0%) changed due to adverse drug reactions such as acute kidney injury, vomiting, or pancytopenia. When antibiotics were changed to second-line antibiotics, the change occurred six days after administering TMP-SMX. (6.36 ± 2.80 days)

We compared the clinical characteristics of the severe PCP group with acute respiratory failure and the non-severe PCP group with low oxygen demand. The severe PCP group was older (41.4 ± 14.7 years vs. 52.2 ± 12.3 years, p = 0.007) and had a higher Body mass index (20.4 ± 2.8 vs. 23.0 ± 3.3, p = 0.005). The type of kidney donation, re-transplantation, and the incidence of prophylaxis failure after transplantation were not significantly different between groups. In the severe PCP group, the time from transplantation (23.4 ± 24.9 months vs. 13.7 ± 9.9 months, p = 0.090) and from discontinuation of prophylaxis (12.8 ± 19.9 months vs. 5.6 ± 2.7 months, p = 0.101) to PCP diagnosis tends to be insignificantly shorter than in the non-severe PCP group. HLA mismatch (2.3 ± 1.1 vs. 3.4 ± 1.7, p = 0.011) was significantly higher, and TLC (970.7 ± 408.3 mm^3^ vs. 571.2 ± 342.7 mm^3^, p = 0.001) was lower in the severe PCP group compared with the non-severe group (Table 1).

Univariate and multivariate logistic regression evaluated risk factors of severe PCP with respiratory failure. As a result, the associated risk factors were age (OR 1.07; 95% CI: 1.01–1.13; p = 0.027), time from transplantation to PCP diagnosis (OR 0.92; 95% CI: 0.86–0.99; p = 0.024), lymphopenia (OR 6.48; 95% CI: 1.05–40.09; p = 0.044), and history of acute rejection within 1 year (OR 8.28; 95% CI: 1.29–53.20; p = 0.026) (Table 2).

**Table 2:** Univariate and multivariate logistic regression for severe PCP

	Univariate analysis		Multivariate analysis	
	Odds ratio (95% CI)	P-value	Odds ratio (95% CI)	P-value
**Age**	1.06 (1.01–1.11)	0.012	1.07 (1.01–1.13)	0.027
**Sex**	1.04 (0.34–3.19)	0.945		
**Time from onset of symptom to hospital admission**	0.90 (0.80–1.01)	0.067		
**Time from transplantation to PCP**	0.97 (0.92–1.01)	0.114	0.92 (0.86–0.99)	0.024
**Time from discontinuation of TMP/SMX to PCP**	0.91 (0.79–1.05)	0.202		
**HLA-A, B, DR mismatch**	1.68 (1.08–2.62)	0.022		
**Immunosuppressant** Tacrolimus Cyclosporin	1.04 (0.31–3.49) 0.96 (0.29–3.24)	0.951 0.951		
**CMV co-infection**	1.57 (0.49–5.08)	0.451		
**BKV co-infection**	1.36 (0.33–5.55)	0.671		
**Use of rituximab**	2.63 (0.79–8.72)	0.114		
**Lymphopenia** (TLC < 1,000 mm^3^)	3.70 (0.96–14.29)	0.058	6.48 (1.05–40.09)	0.044
**History of acute rejection treatment within 1 year**	2.63 (0.79–8.72)	0.114	8.28 (1.29–53.20)	0.026

BKV, BK virus; CI, confidence interval; CMV, cytomegalovirus; HLA, human leukocyte antigen; PCP, pneumocystis pneumonia; TMP/SMX, trimethoprim/sulfamethoxazole

**Fig. 2:**
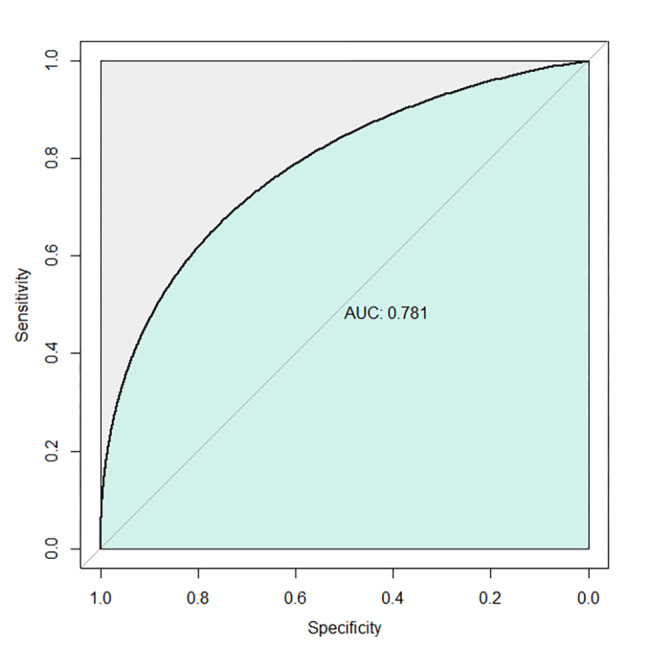
Receiver operating characteristic (ROC) curve of total lymphocyte count (AUC = 0.781).

The cutoff value of 636.4 mm^3^ (Table 3) was obtained using the receiver operating characteristic curve for TLC (Fig. 2).

**Table 3:** Sensitivity and specificity of the cutoff values of total lymphocyte count

	Sensitivity	Specificity	Cutoff value (mm^3^)
**Total lymphocyte count**	0.667	0.870	636.4

## Discussion

Patients with kidney transplantation are vulnerable to a PCP infection, which is a rare but life-threatening opportunistic infection. More than half of patients diagnosed with PCP after kidney transplantation progress to severe PCP with acute hypoxemic respiratory failure [4, 15]. Therefore, understanding the predictors of severe PCP is important to select the cases that need intensive monitoring and treatment.

In our study, 58% of patients were diagnosed with PCP within 1 year of kidney transplantation. Other studies also reported that PCP mainly occurred within 1 year after transplantation [4, 12, 13]. The incidence of PCP is lower within six months after kidney transplantation because TMP-SMX prophylaxis is usually within this period. However, stopping prophylaxis increased the incidence of PCP. In our study, 68% of patients developed PCP within 6 months of stopping TMP-SMX prophylaxis. Kwon et al. suggested that outcomes may be improved by maintaining PCP prophylaxis for up to 1 year in high-risk patients treated with rituximab for desensitization or those receiving acute rejection treatment [10].

As previously discussed, the mortality rate was 30% in severe PCP patients with acute hypoxic respiratory failure. Gaborit B. J. et al. reported that 49.5% of patients with PCP who developed immunosuppression as a result of HIV, hematologic malignancy, or solid organ transplantation, typically had severe PCP that required high-flow oxygen therapy or mechanical ventilation [15]. In our study, limited to kidney transplant patients, 54% of all cases progressed to severe PCP with acute hypoxic respiratory failure, of which 25.9% died. Additionally, old age, the time from a kidney transplant to the diagnosis of PCP, the history of receiving acute rejection treatment within the last 1 year, and the presence of lymphopenia at hospital admission were found to be particularly high-risk factors for acute hypoxic respiratory failure in patients with PCP. Severe PCP was more than eight times more common in this group (OR 8.28 [95% CI: 1.29–53.20], p = 0.026) due to the increased risk of PCP infection brought on by high-dose steroids or intensive immunosuppressive therapy. According to Lee et al., 83.3% of patients infected with PCP following kidney transplantation had a history of acute graft rejection treatment (OR: 11.81 [95% CI: 3.06–45.57], p < 0.001) [14]. A meta-analysis by Seyed et al. examined the risk factors for PCP development after solid organ transplantation [8]. They found that CMV infection (OR: 3.30 [2.07–5.26], p = 0.006) and allograft rejection (OR 2.36 [1.54–3.62], p = 0.05) increased the risk of PCP after organ transplantation, and concluded that extended prophylaxis in patients with acute rejection might reduce the risk of PCP. In our institution, PCP prophylaxis is administered for 6 months after acute rejection treatment, but a shorter outpatient follow-up is needed given the high possibility of these patients developing severe PCP. Additionally, other studies reported CMV co-infection as a risk factor for PCP, but our study did not reveal it as a risk factor for PCP. CMV infection may affect the patient’s immunity, but this study did not indicate significant results.

In our study, lymphopenia at the time of hospital admission was also a predictor of severe PCP progression. Many studies have previously reported that lymphopenia was a predictive factor influencing the development of PCP [9, 21, 22]. We found a difference in TLC between non-severe and severe PCP groups at the time of admission (970.7 ± 408.3 mm^3^ vs. 571.2 ± 342.7 mm^3^, p = 0.001). Additionally, the multiple logistic regression analysis found that lymphopenia was a predictor of progression to severe PCP (OR 6.48 [1.05–40.09], p = 0.044). Given its association with CD4 + count, TLC is a significant risk factor for PCP in patients with HIV. Werbel et al. reported that severe lymphopenia is a risk factor for PCP in solid organ transplant recipients, which increases the risk of PCP development due to recent immunosuppression [21]. Kageyama et al. also reported lower lymphocyte count as a factor affecting PCP prognosis in patients with systemic autoimmune disease [22]. Our study showed similar results, suggesting that lymphopenia reflected a recent immunosuppression state, which is a risk factor for progression to severe PCP.

The first limitation of this study was the possibility of selection bias because it is a retrospective study conducted in a single center. Second, as PCP is rare, the sample size is small. Although the number of cases was larger compared to other studies, the statistical power was inevitably small. So, further prospective data on patients with PCP after kidney transplantation are required.

In conclusion, more than half of the total patients who developed PCP after kidney transplantation progressed to severe PCP with acute hypoxic respiratory failure. Age, history of acute rejection treatment within 1 year, and presence of lymphopenia at the time of admission are risk factors for severe PCP. More aggressive monitoring and treatment are therefore needed in these patients.

## Data Availability

The datasets used and/or analyzed during the current study are available from the corresponding author on reasonable request.

## Abbreviations

PCP: Pneumocystis pneumonia
HIV: Human immunodeficiency virus
CMV: Cytomegalovirus
TMP-SMX: Trimethoprim-sulfamethoxazole
MPD: methylprednisolone
ECMO: Extracorporeal membrane oxygenation
PCR: Polymerase chain reaction
BAL: Bronchoalveolar lavage
TLC: Total lymphocyte count
OR: Odds ratio
CI: Confidence interval
HLA: Human leukocyte antigen

## References

[1] Boonsarngsuk V, Sirilak S, Kiatboonsri S (2009). Acute respiratory failure due to Pneumocystis pneumonia: outcome and prognostic factors. Int J Infect Dis.

[2] Festic E, Gajic O, Limper AH, Aksamit TR (2005). Acute respiratory failure due to pneumocystis pneumonia in patients without human immunodeficiency virus infection: outcome and associated features. Chest.

[3] Monnet X, Vidal-Petiot E, Osman D, Hamzaoui O, Durrbach A, Goujard C (2008). Critical care management and outcome of severe Pneumocystis pneumonia in patients with and without HIV infection. Crit Care.

[4] Hosseini-Moghaddam SM, Shokoohi M, Singh G, Dufresne SF, Boucher A, Jevnikar A (2019). A multicenter case-control study of the effect of acute rejection and cytomegalovirus infection on pneumocystis pneumonia in solid organ transplant recipients. Clin Infect Dis.

[5] Neofytos D, Hirzel C, Boely E, Lecompte T, Khanna N, Mueller NJ (2018). Pneumocystis jirovecii pneumonia in solid organ transplant recipients: a descriptive analysis for the swiss transplant cohort. Transpl Infect Dis.

[6] Garg N, Jorgenson M, Descourouez J, Saddler CM, Parajuli S, Astor BC (2018). Pneumocystis jiroveci pneumonia in kidney and simultaneous pancreas kidney transplant recipients in the present era of routine post-transplant prophylaxis: risk factors and outcomes. BMC Nephrol.

[7] Goto N, Futamura K, Okada M, Yamamoto T, Tsujita M, Hiramitsu T (2015). Management of pneumocystis jirovecii pneumonia in kidney transplantation to prevent further outbreak. Clin Med Insights Circ Respir Pulm Med.

[8] Hosseini-Moghaddam SM, Krishnan RJ, Guo H, Kumar D (2018). Cytomegalovirus infection and graft rejection as risk factors for pneumocystis pneumonia in solid organ transplant recipients: a systematic review and meta-analysis. Clin Transpl.

[9] Iriart X, Challan Belval T, Fillaux J, Esposito L, Lavergne RA, Cardeau-Desangles I (2015). Risk factors of pneumocystis pneumonia in solid organ recipients in the era of the common use of posttransplantation prophylaxis. Am J Transplant.

[10] Kim YH, Kim JY, Kim DH, Ko Y, Choi JY, Shin S (2020). Pneumocystis pneumonia occurrence and prophylaxis duration in kidney transplant recipients according to perioperative treatment with rituximab. BMC Nephrol.

[11] Gordon SM, LaRosa SP, Kalmadi S, Arroliga AC, Avery RK, Truesdell-LaRosa L (1999). Should prophylaxis for pneumocystis carinii pneumonia in solid organ transplant recipients ever be discontinued?. Clin Infect Dis.

[12] Kim JE, Han A, Lee H, Ha J, Kim YS, Han SS (2019). Impact of Pneumocystis jirovecii pneumonia on kidney transplant outcome. BMC Nephrol.

[13] Radisic M, Lattes R, Chapman JF, del Carmen Rial M, Guardia O, Seu F (2003). Risk factors for pneumocystis carinii pneumonia in kidney transplant recipients: a case-control study. Transpl Infect Dis.

[14] Lee SH, Huh KH, Joo DJ, Kim MS, Kim SI, Lee J (2017). Risk factors for pneumocystis jirovecii pneumonia (PJP) in kidney transplantation recipients. Sci Rep.

[15] Gaborit BJ, Tessoulin B, Lavergne RA, Morio F, Sagan C, Canet E (2019). Outcome and prognostic factors of pneumocystis jirovecii pneumonia in immunocompromised adults: a prospective observational study. Ann Intensive Care.

[16] Kotani T, Katayama S, Miyazaki Y, Fukuda S, Sato Y, Ohsugi K (2017). Risk factors for the mortality of pneumocystis jirovecii pneumonia in non-HIV patients who required mechanical ventilation: a retrospective case series study. BioMed Res Int.

[17] Ko RE, Na SJ, Huh K, Suh GY, Jeon K (2019). Association of time-to-treatment with outcomes of pneumocystis pneumonia with respiratory failure in HIV-negative patients. Respir Res.

[18] Salzer HJF, Schafer G, Hoenigl M, Gunther G, Hoffmann C, Kalsdorf B (2018). Clinical, diagnostic, and treatment disparities between HIV-infected and non-HIV-infected immunocompromised patients with pneumocystis jirovecii pneumonia. Respiration.

[19] Ding L, Huang H, Wang H, He H (2020). Adjunctive corticosteroids may be associated with better outcome for non-HIV pneumocystis pneumonia with respiratory failure: a systemic review and meta-analysis of observational studies. Ann Intensive Care.

[20] Ko Y, Jeong BH, Park HY, Koh WJ, Suh GY, Chung MP (2014). Outcomes of pneumocystis pneumonia with respiratory failure in HIV-negative patients. J Crit Care.

[21] Werbel WA, Ison MG, Angarone MP, Yang A, Stosor V (2018). Lymphopenia is associated with late onset pneumocystis jirovecii pneumonia in solid organ transplantation. Transpl Infect Dis.

[22] Kageyama T, Furuta S, Ikeda K, Kagami SI, Kashiwakuma D, Sugiyama T (2019). Prognostic factors of pneumocystis pneumonia in patients with systemic autoimmune diseases. PLoS ONE.

